# Serum Ghrelin Concentration in Patients With Primary Biliary Cirrhosis (PBC)

**DOI:** 10.7759/cureus.20288

**Published:** 2021-12-09

**Authors:** Rania Naguib, Amel Fayed, Eman Elkemary, Hend Naguib

**Affiliations:** 1 Clinical Science Department, College of Medicine, Princess Nourah Bint Abdulrahman University, Riyadh, SAU; 2 Endocrinology Unit, Internal Medicine Department, Faculty of Medicine, Alexandria University, Alexandria, EGY; 3 Clinical Pathology Department, Faculty of Medicine, Alexandria University, Alexandria, EGY; 4 Hepatology Unit, Internal Medicine Department, Faculty of Medicine, Alexandria University, Alexandria, EGY

**Keywords:** primary biliary cholangitis, child–pugh (c-p) classification, severity, primary biliary cirrhosis (pbc), ghrelin

## Abstract

Introduction: The appetite-modulating hormone ghrelin may have a role in the etiology of anorexia which is a serious concern in patients with primary biliary cirrhosis (PBC). This study aims to assess the difference in ghrelin level between cases of PBC and healthy controls matched for age and gender, and to evaluate the level of ghrelin in relation to clinical and laboratory findings among cases.

Methods: Twenty patients with primary biliary cirrhosis and 30 healthy controls matched by gender and age were recruited. The severity of liver disease was determined using the Child-Pugh grading system. Clinical comorbidities such as a history of ascites, gastrointestinal bleeding, and encephalopathy were evaluated. A commercial enzyme-linked immunosorbent assay was used to measure total ghrelin.

Results: PBC cases had a significantly higher average level of ghrelin (2305.3 ± 639.4) pg/mL compared to controls (682 ± 197.3) pg/mL. Furthermore, the minimum reported level in cases was 1258 pg/mL compared to 326 pg/mL in controls, while the maximum level nearly tripled the control’s maximum level. In PBC patients, plasma levels of total ghrelin showed a weak positive correlation with age, an inverse correlation with body mass index, and were not associated with gender. The level was significantly higher than those in the controls. Ghrelin was associated with the severity of cirrhosis. Levels of serum ghrelin were higher in patients with associated comorbidities such as a history of ascites, gastrointestinal bleeding, and encephalopathy.

Conclusions: Our study demonstrated elevated serum ghrelin levels in patients with primary biliary cirrhosis. Serum ghrelin was associated with the degree of severity and the presence of related comorbidities. Patients with primary biliary cirrhosis remain anorexic and catabolic despite elevated ghrelin levels, suggesting tissue resistance to this anabolic peptide which could be crucial to understanding anorexia and cachexia in primary biliary cirrhosis.

## Introduction

Gut hormones are involved in food intake and energy homeostasis on several levels including central appetite regulation and regulation of gastrointestinal motility [1]. Ghrelin is a cytokine produced mostly by the stomach fundus and is considered the only established peripheral hormone with an orexigenic function that has been discovered so far. Ghrelin stimulates appetite and food intake and is important for regulating metabolism and energy balance [2-4]. Its concentration rises pre-prandially, helping to initiate meals [5]. It has a diabetogenic effect, inhibiting insulin release and stimulating glucose output by hepatocytes, as well as strong growth hormone (GH) releasing activity. It also has lipogenic and antilipolytic properties and anti-inflammatory effects [6,7]. In humans, ghrelin infusion resulted in a short-term increase in hunger [8]. Fasting ghrelin concentrations are higher in anorexia and cachexia in patients suffering from chronic illnesses but lower in obesity indicating a compensatory mechanism. The orexigenic properties of ghrelin and its potential clinical usage to enhance appetite to compensate for anorexia-cachexia seen in patients with chronic illnesses have been investigated [9-11]. Ghrelin increases appetite in the short term by targeting the melanocortin system in the hypothalamic arcuate nucleus and increases adiposity in peripheral tissues in the long term [12].

Ghrelin binds to the growth hormone secretagogue receptor 1a (GHSR1a), which has been identified as an important partner in GH release and appetite stimulation [12,13]. GHSR1a is found in a variety of metabolically active tissues, including the pancreas, liver, heart, and gonads, as well as at the central level in the hypothalamus and pituitary [14]. This broad expression could explain why GHSR1a is so important for glucose homeostasis in the presence of negative energy balance [7,12]. It has been suggested in recent years that ghrelin’s principal function is to maintain glucose levels in times of food scarcity [9]. In adults with cirrhosis, total fasting ghrelin levels have been found to be either low, normal, or increased and the findings of previous studies are controversial [15-17]. Malnutrition and anorexia are common in cirrhotic patients and their mechanisms are not fully understood [17]. Early satiety associated with large-volume ascites or hepatosplenomegaly may be followed by reduced food intake. Anorexia can also be caused by a restricted diet, poor palatability of food. Malnutrition could also be attributed to abnormalities in absorption and digestion, along with the reduced hepatic synthesis of energy substrates [15,16].

Primary biliary cholangitis, formerly known as primary biliary cirrhosis, is an autoimmune progressive liver disease that primarily affects women (with an incidence of 20 to 40 cases per 100,000) [7,18-20]. It is characterized by inflammation of the small and medium-sized bile ducts, destruction of interlobular bile ductules, cholestasis, cirrhosis, end-stage liver disease, and death [21-23]. The mean survival is 9.17 years in the presence of cirrhosis and 10.7 years in the absence of cirrhosis. PBC is responsible for 2.2% of all deaths caused by liver cirrhosis. Skin itching accompanied by a sleep disorder, debilitating exhaustion, as well as malabsorption of fats and fat-soluble vitamins might contribute to the slowly progressive weight loss and malnutrition in patients with PBC [23]. Despite its importance in food intake, energy balance, and the regulation of the growth hormone-releasing mechanism, ghrelin's role in PBC has yet to be well investigated.

This study aims to assess the difference in ghrelin level between cases of PBC and healthy controls matched for age and gender and to evaluate the level of ghrelin in relation to clinical and laboratory findings among cases.

## Materials and methods

Study design

This is a case-control study that was conducted during the period between February and June 2021. The study was approved by the Institutional Ethics and Research Committee at Alexandria Faculty of Medicine, Alexandria, Egypt, with an approval number 21-389. Written informed consent was taken from all participants. Twenty patients diagnosed with PBC were recruited using convenient sampling from Alexandria Main University Hospital, Internal Medicine Department, Hepatology outpatient clinic.

Inclusion and exclusion criteria

The inclusion criterion was patients with PBC with age at least 18 years. Diagnosis of PBC was based on biochemical evidence of cholestasis, consistent histological findings, antimitochondrial antibody M2 positivity, and no extrahepatic cholestasis. Patients having other chronic disorders (endocrine, renal, respiratory, cardiac, or neurologic), any malignancy (including hepatocellular carcinoma), any current acute infection, hepatic encephalopathy, hepatorenal syndrome, or untreated thyroid dysfunction were excluded.

Data collection

For each patient, relevant demographic and clinical data were collected and recorded. A structured questionnaire was given to all patients to investigate the probable diagnosis of ascites, history of any gastrointestinal tract (GIT) bleeding, and encephalopathy. Thirty healthy age and sex-matched people with normal medical histories, physical examinations, and blood chemistry results were selected based on convenient sampling served as the control group. The body mass index (BMI) was calculated by dividing the weight in kilograms by the square of height in meters (Kg/m^2^). The BMI was calculated in patients with ascites using their weight prior to the onset of ascites or their body weight following therapeutic paracentesis. After an overnight fast, venous blood was obtained in the morning for biochemical analysis as part of standard clinical follow-up. Serum albumin, bilirubin, prothrombin activity as well as the presence of ascites and grade of encephalopathy were assessed and Child-Pugh (C-P) classification [15] was used to determine the severity of the liver disease.

Measurement of serum ghrelin

To measure ghrelin, venous blood samples were collected between 8-9 AM after overnight fasting. Serum was allowed to clot for 10-20 minutes, immediately separated by centrifugation for 20 minutes at 2000-3000 revolutions per minute (RPM), and then stored at -80°C until analysis. The concentration of ghrelin was determined using a sandwich Enzyme-Linked Immunosorbent Assay (ELISA) kit (Bioassay Technology Laboratory, Shanghai, China), which uses plate pre-coated antibody specific for the human ghrelin, biotinylated human ghrelin antibody, and streptavidin- HRP (a tetrameric biotin-binding protein). It has a sensitivity of 10 pg/mL (lowest detection limit). The ELISA microplate reader used was Stat Fax 2100 (Fisher Bioblock Scientific, USA) and to minimize individual variations, serum levels were determined in duplicate [24]. The group of PBC patients was compared to the control group regarding serum total ghrelin concentration and the correlation with different parameters was studied.

Statistical analysis

Data were analyzed using the IBM SPSS software package, version 20.0 (IBM Corp., Armonk, NY). Student t-test/Mann-Whitney tests were used to compare two groups for quantitative variables while ANOVA/Kruskal-Wallis tests were used for comparing the different Child classes after verifying the normality distribution of variables. The Pearson correlation coefficient was used to correlate between ghrelin level and both age and BMI. A multivariate linear regression model was developed with ghrelin level as an outcome and both PBC and BMI as covariates to test the independent relation between PBC and ghrelin level. A p-value less than 0.05 was considered as statistically significant.

## Results

The current study involved a total of 20 PBC cases and 30 controls. Cases and controls were matched for age and gender. The mean age was 60.2±2.8 years and the majority of participants were females (43, 86%) with an average BMI of 24.1±4.1 Kg/m^2^. Cases had significantly lower BMI (19.8 ± 2.6 Kg/m^2^) compared to controls (27 ± 1.52.6 Kg/m^2^).

Out of the 20 PBC cases, four patients (20%) were classified as Child A and seven (35%) as Child C. History of ascites was confirmed in 80% of PBC cases (n=16), encephalopathy in 65% (n=13), and GI bleeding was verified in 55% of cases (11 patients) (Table 1).

**Table 1:** Distribution of the studied cases according to demographic data in the studied sample BMI = Body mass index

Characteristics	Total (N=50)	Cases (N=20)	Control (N=30)
Age (Years)			
Mean ± SD	60.2 ± 2.8	60.6 ± 3.3	60 ± 2.4
Median (Min. – Max.)	60 (55 – 67)	60 (56 – 67)	60 (55 – 64)
Gender			
Male	7 (14%)	3 (15%)	4 (13.3%)
Female	43 (86%)	17 (85%)	26 (86.7%)
BMI (Kg/m^2^)			
Mean ± SD	24.1 ± 4.1	19.8 ± 2.6	27 ± 1.5
Median (Min. – Max.)	25 (16 – 29)	19.5 (16 – 26)	27 (24 – 29)
Child Class (n=20)			
A		4 (20%)	
B		9 (45%)	
C		7 (35%)	
Ascites (n=20)		16 (80%)	
Encephalopathy (n=20)		13 (65%)	
Bleeding (n=20)		11 (55%)	

**Figure 1:**
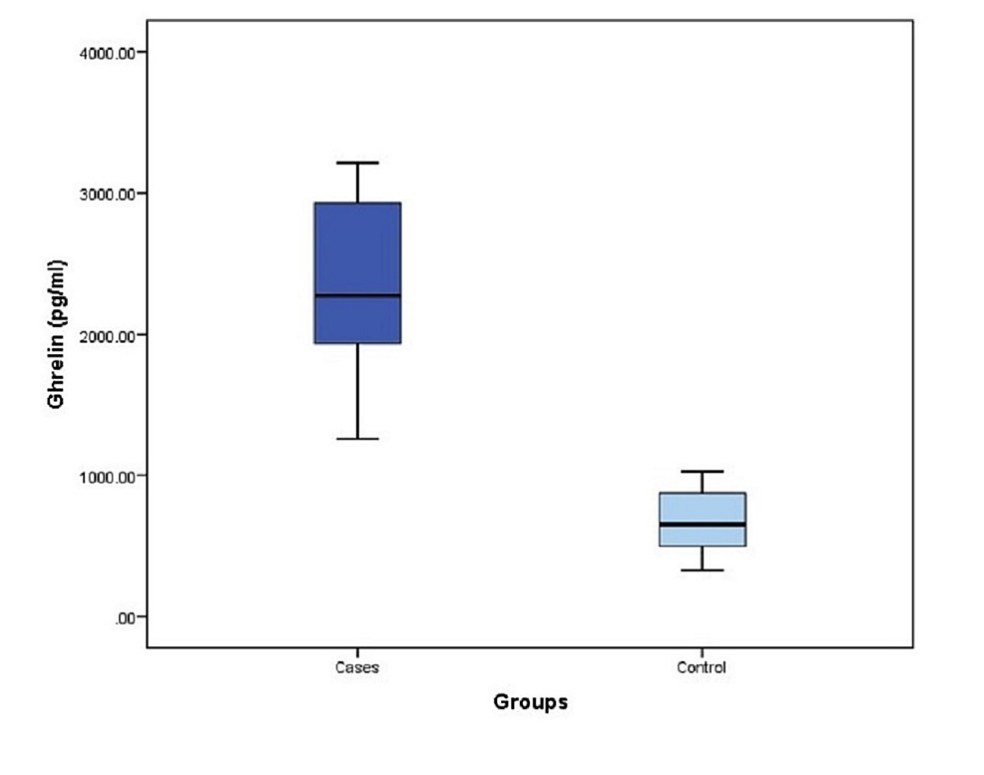
Distribution of ghrelin levels among cases versus controls

**Figure 2:**
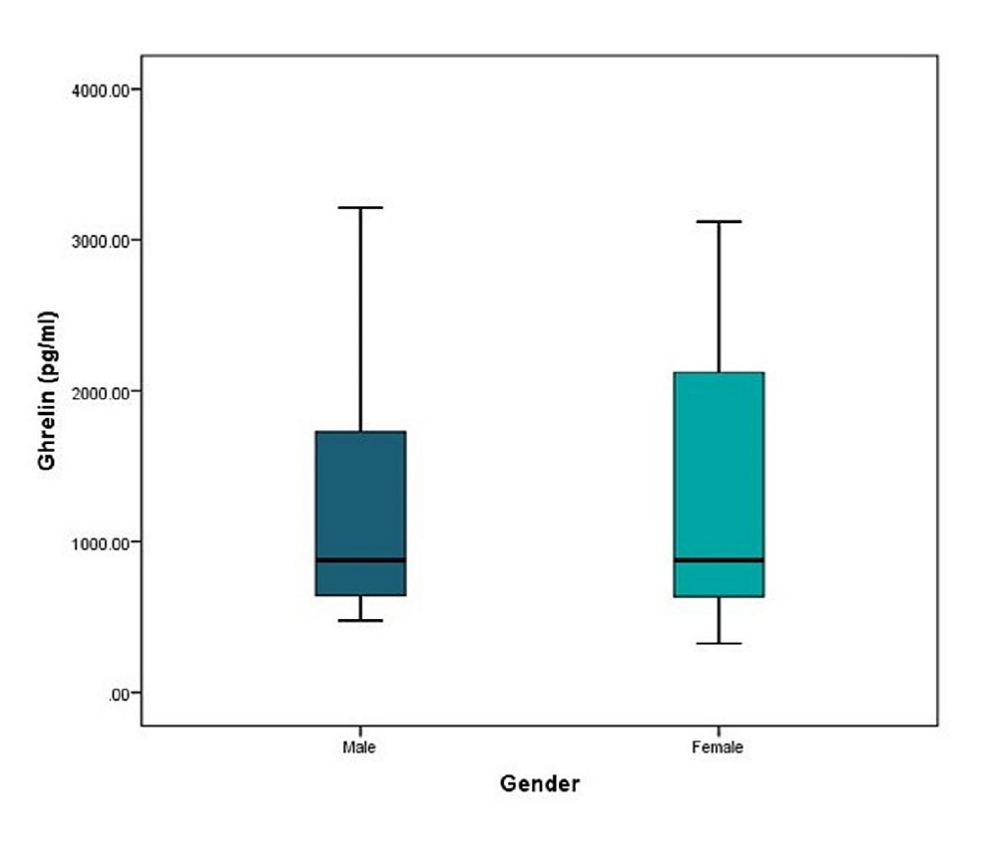
Distribution of ghrelin levels among males versus females

PBC cases had a significantly higher average level of ghrelin (2305.3 ± 639.4) pg/mL compared to controls (682 ± 197.3) pg/mL. Furthermore, the minimum reported level in cases was 1258 pg/mL compared to 326 pg/mL in controls, while the maximum level nearly tripled the control’s maximum level. Ghrelin levels did not differ significantly between males and females (Figures 1, 2).

Ghrelin level was trending up across patients in different Child classes; the lowest level was observed in patients with Child A (1341 ± 82.9) pg/mL, followed by Child B (2181.7 ± 184) pg/mL and the highest in Child C patients (3015.3 ± 123.1) pg/mL.

When comparing PBC patients who had a positive history of associated comorbidities to those who did not, the level of ghrelin was consistently greater. Patients who had ascites had nearly double ghrelin (2546.4 ± 454.5) pg/mL level compared to those without ascites (1341 ± 82.9 pg/mL), those who had encephalopathy averaged 2648.4 ± 440.9 pg/mL versus 1668.1 ± 421.1 for those without encephalopathies, and patients with a history of bleeding showed considerably higher levels of ghrelin (2682.2 ± 408.5) pg/mL compared to patients without a history of bleeding (1844.7 ± 573.1 pg/mL) (Table 2).

**Table 2:** Difference in ghrelin levels among cases by Child classification and diseases associated morbidity ¶ ANOVA test, ‡ t-test

	No.	Ghrelin (pg/mL)		p
		Mean ± SD.	Median (Min. – Max.)	
Child class				
A	4	1341 ± 82.9	1325 (1258 – 1456)	<0.001¶
B	9	2181.7 ± 184	2134 (1923 – 2453)	
C	7	3015.3 ± 123.1	3002 (2876 – 3214)	
Ascites				
No	4	1341 ± 82.9	1325 (1258 – 1456)	<0.001‡
Yes	16	2546.4 ± 454.5	2421 (1923 – 3214)	
Encephalopathy				
No	7	1668.1 ± 421.1	1456 (1258 – 2236)	<0.001‡
Yes	13	2648.4 ± 440.9	2876 (1923 – 3214)	
Bleeding				
No	9	1844.7 ± 573.1	1923 (1258 – 3002)	0.001‡
Yes	11	2682.2 ± 408.5	2876 (2109 – 3214)	

**Figure 3:**
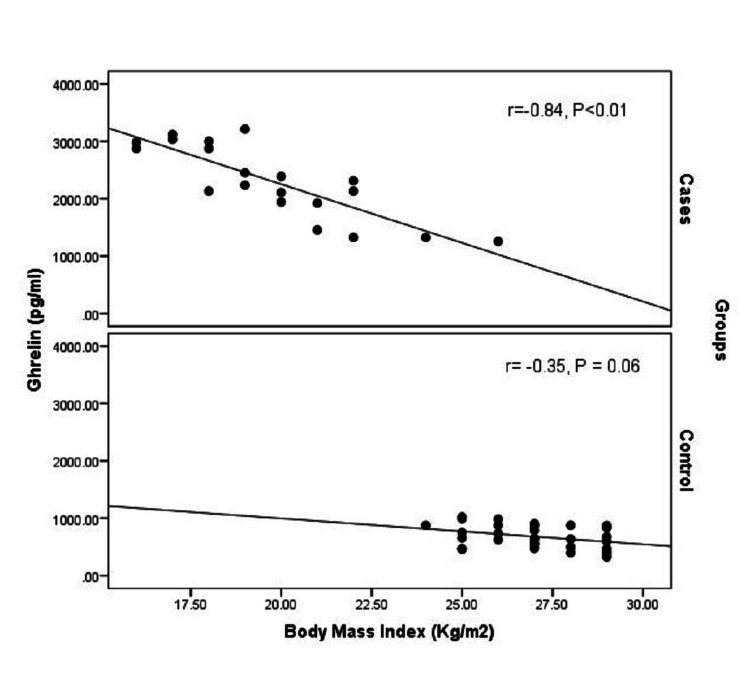
Correlation between ghrelin level and body mass index (BMI) among cases and controls

**Figure 4:**
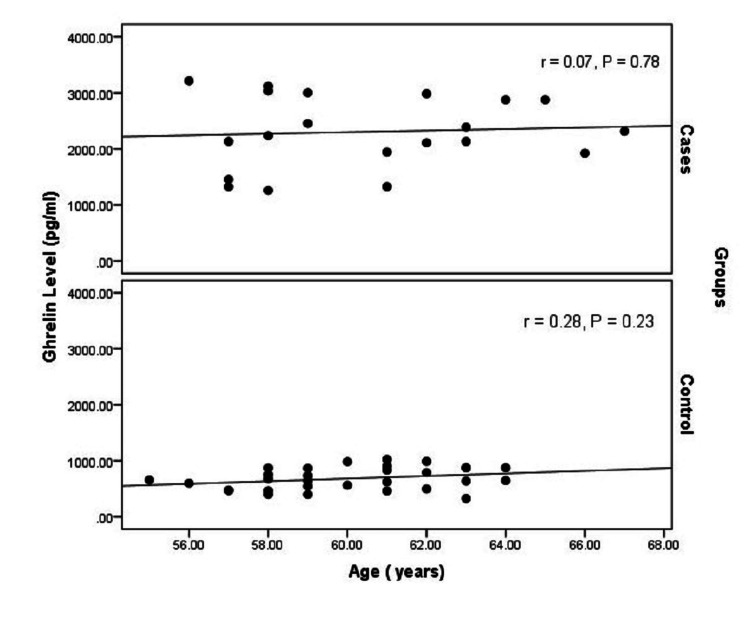
Correlation between ghrelin level and age among cases and controls

An inverse significant correlation was confirmed between ghrelin level and BMI; the higher the BMI, the lower the ghrelin level (r=-0.932, P<0.01). On the contrary, a weak positive correlation was noted between the ghrelin level and age; the older the patient, the higher the ghrelin level (r=0.136, p=0.34) (Figures 3, 4).

A multivariate linear regression model was used to investigate the independent relation between ghrelin and PBC while adjusting for the BMI. Both PBC and BMI were significantly correlated to ghrelin level; PBC will have a higher ghrelin level by an average of 546.7 pg/mL (Beta coefficient = 546.7, p<0.01) while, increasing BMI by 1 Kg/m^2^ will decrease the ghrelin level by an average of 149.2 pg/mL (Beta coefficient = -149.2, p<0.01).

## Discussion

The appetite-modulating hormone ghrelin may have a role in the pathogenesis of anorexia in patients with PBC. In this study, fasting ghrelin level was assessed in patients with a wide range of hepatic dysfunction as indicated by the C-P classification and compared to normal control subjects. The relation between serum ghrelin and the presence of history of ascites, GIT bleeding, and encephalopathy was evaluated. A significant negative correlation was confirmed between ghrelin level and BMI. This result was reported by previous studies [16,17,25]. This denotes that serum ghrelin level depends on the nutritional status and can be used as a marker to help with anorexia and enhance dietary supplements for patients with liver cirrhosis. Malnutrition and hypermetabolism are common in cirrhotic patients and are well-known risk factors for poor prognosis [17]. Ghrelin, as an early indicator of malnutrition, can also be used as a predictive factor for survival and prognosis. A weak positive correlation was noted between serum ghrelin and age whereas ghrelin level was not associated with gender. A similar study confirmed the positive correlation between age and serum ghrelin and the lack of association with gender but failed to prove any correlation with BMI [15].

In the present study, serum ghrelin levels were higher in patients with PBC compared to control subjects. Fasting plasma ghrelin levels in cirrhotic patients have been studied in different reports and was found to be controversial. It has been described as being normal [26], raised [16,27-29], or lowered [30,31] compared to control subjects. Different patients’ criteria, control subject selection, or both could explain the disparities. Some of the patients had malignancies and were not BMI-matched to the control group [17].

In normal circumstances, ghrelin increases appetite and food intake and plays a crucial role in meal initiation. Insulin may play a role in the dysregulation of ghrelin. In humans, insulin has been proven to have a direct suppressive effect on serum ghrelin [16,31]. Hyperinsulinemia associated with insulin resistance in individuals with cirrhosis, on the other hand, can cause the previously noted increased pattern of ghrelin secretion. Even though ghrelin does not appear to be a cause of malnutrition, increased ghrelin production in cirrhotic individuals may reflect an adaptive mechanism signaling the hypothalamus to stimulate appetite and maintain energy balance in response to their poor nutritional state. These patients with a lack of appetite appeared to be resistant to ghrelin's orexigenic effects. Increased ghrelin and decreased feeding, leading to malnutrition, could be explained in part by desensitization of the hypothalamic ghrelin receptor which is important in the control of food intake. The effects of exogenous ghrelin have been shown to be reduced in individuals with anorexia nervosa who have been exposed to hyperghrelinemia for a long time [16,32]. Furthermore, in the hypothalamus, ghrelin receptor mRNA levels, as well as ghrelin gene expression and peptide content have been demonstrated to be significantly suppressed in response to altered nutritional conditions such as fasting [33,34]. It has been claimed in recent years that the major function of ghrelin is to maintain glucose levels in times of food scarcity by regulating GH secretion [7]. This might be an adaptive compensatory mechanism in patients with cirrhosis to protect against hypoglycemia.

In our study, ghrelin level was associated with the severity of cirrhosis. Child C patients had considerably greater serum ghrelin levels compared to both Child A and B and control groups. These findings are consistent with those of Ataseven et al. [35], Tacke et al. [27], El-Shehaby et al. [16], and Elbadri et al. [17] who reported that plasma ghrelin levels were higher in cirrhotic individuals with Child C. These findings contradicted those of Marchesini et al. [26] and Kalaitzakis et al. [36] who found that plasma ghrelin levels were unrelated to the severity of the liver disease. The rise in plasma ghrelin in response to these alterations is probably through different mechanisms. It can be explained by the fact that metabolic decompensation and clinical consequences such as malnutrition increase with Child's class. This would result in GH release, hyperglycemia induction, energy balance modification, hunger stimulation, and thus an increase in food intake. Another explanation of the association of serum ghrelin level with the severity of liver disease is that the advanced disease in Child C is linked to severe hepatic failure, cachexia, endotoxemia, and hemodynamic abnormalities, all of which might affect cytokine and vasoactive substance levels in the blood. A third explanation is a hypothesis, which is not tested in our study is that micronutrient deficiencies or toxic by-products of protein breakdown in cirrhosis may harm appetite-regulating hypothalamic neurons, resulting in altered ghrelin sensitivity [16].

Levels of serum ghrelin were higher in patients with associated comorbidities such as the presence of ascites, history of GIT bleeding, and encephalopathy. These results are in accordance with previous pieces of literature [16,17,27]. Increased ghrelin levels have a direct effect on hemodynamic parameters and capillary function, which could explain why it is associated with clinical symptoms like encephalopathy in people with liver cirrhosis [27].

Up to our knowledge, this is the first study to report that plasma ghrelin levels are increased in patients with PBC compared to control. Only one study was performed on PBC patients in 2004 [31] and it stated that plasma ghrelin level is lower in patients with PBC compared to control. This finding is contradictory to our results, but the difference might be attributed to the presence of different subtypes of ghrelin and the selection criteria in their study since their patients were all C-P class A. 

We should, however, acknowledge the limitation that we did not assess acyl-ghrelin, although there are issues regarding the specificity of existing acyl-ghrelin assays [30]. Although total ghrelin has a well-established link with metabolic disturbances, acylated ghrelin has been proven to be particularly relevant in the development of metabolic abnormalities. In fact, the majority of studies investigating the link between metabolic abnormalities and ghrelin have focused on total ghrelin estimation [7]. Another limitation of our study is the small sample size of the studied group.

## Conclusions

In conclusion, our study demonstrated elevated serum ghrelin levels in patients with PBC. Furthermore, serum ghrelin was associated with the degree of severity and the presence of related comorbidities. Patients with PBC remain anorexic and catabolic despite elevated ghrelin levels, suggesting tissue resistance to this anabolic peptide which could be crucial to understanding anorexia and cachexia in PBC. Further, larger clinical studies are needed to confirm the results of the current study. Assessing the active form of ghrelin, acyl-ghrelin, rather than total ghrelin is recommended. More studies are needed to clarify the mechanism of increased ghrelin in patients with PBC and to assess the ghrelin receptor sensitivity mechanisms in the hypothalamus as a probable cause of malnutrition in those patients.
